# New perspectives on cytoskeletal dysregulation and mitochondrial mislocalization in amyotrophic lateral sclerosis

**DOI:** 10.1186/s40035-021-00272-z

**Published:** 2021-11-15

**Authors:** Frances Theunissen, Phillip K. West, Samuel Brennan, Bojan Petrović, Kosar Hooshmand, P. Anthony Akkari, Matt Keon, Boris Guennewig

**Affiliations:** 1 Perron Institute for Neurological and Translational Science, Nedlands, WA Australia; 2 Centre for Molecular Medicine and Innovative Therapeutics, Murdoch University, Murdoch, WA Australia; 3 GenieUs Genomics Pty Ltd, Sydney, NSW Australia; 4 Brain and Mind Centre and Central Clinical School, Faculty of Medicine and Health, the University of Sydney, Camperdown, NSW Australia; 5 Division of Neurology, Duke University Medical Centre, Duke University, Durham, NC USA

**Keywords:** Cytoskeleton, Neurofilament, Mitochondria, Amyotrophic lateral sclerosis, Neurodegeneration, Axonal transport, Gut microbiome

## Abstract

Amyotrophic lateral sclerosis (ALS) is a progressive neurodegenerative disease characterized by selective, early degeneration of motor neurons in the brain and spinal cord. Motor neurons have long axonal projections, which rely on the integrity of neuronal cytoskeleton and mitochondria to regulate energy requirements for maintaining axonal stability, anterograde and retrograde transport, and signaling between neurons. The formation of protein aggregates which contain cytoskeletal proteins, and mitochondrial dysfunction both have devastating effects on the function of neurons and are shared pathological features across several neurodegenerative conditions, including ALS, Alzheimer's disease, Parkinson's disease, Huntington’s disease and Charcot-Marie-Tooth disease. Furthermore, it is becoming increasingly clear that cytoskeletal integrity and mitochondrial function are intricately linked. Therefore, dysregulations of the cytoskeletal network and mitochondrial homeostasis and localization, may be common pathways in the initial steps of neurodegeneration. Here we review and discuss known contributors, including variants in genetic loci and aberrant protein activities, which modify cytoskeletal integrity, axonal transport and mitochondrial localization in ALS and have overlapping features with other neurodegenerative diseases. Additionally, we explore some emerging pathways that may contribute to this disruption in ALS.

## Introduction

Neurons have unique and specialized morphological features that enable them to receive, process and transmit information [1]. Since axons can extend up to one meter in length in humans, the presence of both the neuronal cytoskeleton and mitochondria is therefore essential to establish and maintain the polarity and the physiological properties of these enormous structures [1, 2]. The neuronal cytoskeleton is composed of actin filaments, intermediate filaments and microtubules that facilitate the transmission of electrical and chemical signals between neurons and regulate the balance between motility and stability in neuronal structures [3]. To maintain neural connections, correct localization of mitochondria is essential due to the central role of these organelles in adenosine triphosphate (ATP) production, metabolite synthesis, calcium homeostasis and local protein synthesis [4, 5]. Interactions between mitochondria and cytoskeletal motor proteins are therefore critical to establish long-distance transport and positioning of mitochondria along the axon and within dendrites [2]. Changes in the levels, dynamics and stability of cytoskeletal proteins, as a result of mutations or alterations in molecular pathways involved in axonal transport, are associated with the formation of protein aggregates in neurons and glia of the central nervous system (CNS) [3, 6]. Protein aggregates are an established cytopathological feature present in 97% of amyotrophic lateral sclerosis (ALS) patients [7, 8] and are a shared pathological hallmark of Alzheimer's disease (AD), Parkinson's disease (PD), Huntington's disease (HD) and numerous other neurodegenerative diseases [9]. These findings suggest a pathogenic link that connects the initial steps of neurodegenerative processes to the dysregulated transport of vesicles and cargos (mRNA and growth factors), impaired mitochondrial trafficking, and neurofilament accumulation within the axon [10].

Here, we discuss the delicate balance between cytoskeletal integrity (for maintaining transport networks and axonal diameter) and mitochondrial dynamics (for maintaining adequate energy production and mitochondrial integrity for facilitating axonal transport). Since cytoskeletal dysregulation and mitochondrial mislocalization are common features in ALS and several other neurodegenerative diseases, it is important to understand how one affects the other and vice versa. Evidently, it is important to explore new molecular pathways that underpin this relationship, which could be used as novel therapeutic targets or to provide insight into common pathways involved in the neurodegenerative process.

## Neuronal intracellular transport during homeostasis

Axonal transport in healthy neurons is central to maintain intracellular homeostasis, as well as their interactions with neighboring neurons. Specialized transport mechanisms are therefore necessary for neurons to efficiently move molecules from the soma along the length of an axon [11–15]. Transport within axons occurs in a bidirectional manner. Anterograde translocation moves newly synthesized mRNA and proteins such as neurotransmitters, precursors, and enzymes towards the end of axon. On the other hand, retrograde translocation moves cytotoxic metabolites generated in the axonal terminal, as well as aged or damaged proteins and organelles targeted for degradation and recycling, towards the neuron cell body [13]. There are two modes of axonal transport: the fast component, where molecules move at a rate measurable in microns per second, and the slow component, where molecules move at a fraction of a micron per minute [16]. Both modes of transport depend on the specific organization of the cytoskeleton and consume large amounts of ATP. Mitochondria are the major ATP-producing organelles of the cell and are therefore required for axonal transport but are also subject to axonal transport themselves.

### Cytoskeletal structures facilitating neuronal transport

The cytoskeleton is formed by a three-dimensional scaffold of proteins that maintain the structural integrity of the neuron across its different cellular compartments. During the transmission of electrical and chemical signals along and between neurons, the cytoskeleton must be both dynamic and stable in order to facilitate the proper operation of the neuronal circuitry. The cytoskeleton plays an instrumental role in many conditions, including growth and guidance of axons during embryonic development, in maintenance and plasticity of mature neurons and during the regeneration of peripheral axons following injury [3]. The neuronal cytoskeleton has three major components, including microtubules (24 nm in diameter), neurofilaments (intermediate filaments, 10 nm in diameter) and microfilaments (actin-based filaments, 6 nm in diameter) [3]. The microtubules are organized into tubular structures and establish pathways for vesicular, mitochondrial and mRNA transport along the axons and in growth cones to promote neuronal homeostasis [3]. The neurofilaments form the structural core and adjust the diameter of axons, thereby maintaining axonal transport and signal transduction [17]. Finally, the microfilaments contribute to the scaffolding of dynamic structures in mature neurons, such as the lamellipodia and filopodia of neuronal growth cones [3].

In mature neurons, the intermediate type IV filaments are the major cytoskeletal component. The neurofilament structure consists of homo- and heteropolymers made up of combinations of neurofilament light (NEFL, encoded by *NEFL*), neurofilament medium (NEFM, encoded by *NEFM*), and neurofilament heavy (NEFH, encoded by *NEFH*), with internexin-alpha (INA, encoded by *INA*) in the CNS and its analogue from the type III filament group, peripherin (PRPH, encoded by *PRPH*), in the peripheral nervous system [3]. Importantly, these proteins require a specific stoichiometry that is critical for the assembly of the mature neurofilament structure. Each neurofilament protein undergoes post-translational modifications, such as phosphorylation and glycosylation that are critical for its interaction with other filaments during assembly. For instance, phosphorylation of the head domain of neurofilament proteins sustains their disassembled state so that they can be moved along the microtubules *via* slow axonal transport [18]. Following transport, the phosphate moieties on the head domain are removed and the tail domains of NEFM and NEFH are extensively phosphorylated, allowing the filaments to assemble with a light:medium:heavy ratio of about 4:2:1 [19, 20]. Once assembled, the intermediate filaments bind, direct and position mitochondria and other organelles throughout the cell [21]. For instance, the side arms on NEFM and NEFH interact with mitochondria and immobilize them along with the cytoskeletal network [21].

### Axonal transport machinery

Microtubules are the essential tracks upon which molecular cargos are shuttled along the axon in both anterograde and retrograde directions [22]. Axonal transport requires three basic components: the microtubule network, molecular motors and various adaptor proteins that actively shuttle cargos along the axon cytoskeleton [23]. The microtubule structure consists of alpha- and beta-tubulin heterodimers that assemble in a head-to-tail fashion, forming proto-filaments that wrap around one another to create a cylindrical structure [22]. Microtubule networks are polar and uniform in their orientation, with the fast-growing (+) ends projecting toward the synapse, while the stable, slow-growing (−) ends are directed toward the cell body. The microtubule-based molecular motors recognise the intrinsic polarity of microtubules, selectively moving cargos towards their (+) or their (−) ends [24].

The microtubule-based motors are a class of proteins that contain a microtubule-binding sequence and an ATP-binding sequence. These motors drive intracellular trafficking by hydrolysing ATP to generate movement along cytoskeletal microtubules [25]. Kinesins are a large family of 45 proteins that form the molecular motors responsible for anterograde axonal transport directed by the (+) end of the microtubule [26]. The kinesin-1 motor consists of a kinesin heavy chain dimer, encoded by *KIF5A*, *KIF5B* and *KIF5C*, as well as a dimer of kinesin light chains [27]. Kinesin motors facilitate the anterograde transport of synaptic vesicles, mitochondria, ion channels, adhesion molecules and mRNA from the cell body toward synaptic terminals and into the growth cone [13, 25]. Conversely, dynein molecular motors are responsible for retrograde transport. The dynein motor complex consists of two heavy chains (encoded by *DYNC1H1/2*), two intermediate chains, two intermediate light chains and several light chains [26]. Dynein-1 is the major retrograde motor in axons and is responsible for transporting signaling endosomes containing neurotrophins and other factors, autophagosomes, and injury signals toward the cell body for degradation [13]. However, most cargos, including mitochondria, certain endosomal populations, lysosomes and mRNAs, have both motor types simultaneously bound and can be transported in a bi-directional manner [13, 14].

In order to assist motor proteins with cargo binding, motility and localization along the axon, the presence of adaptor proteins is required. For instance, the adaptor proteins Miro and Milton are necessary to anchor mitochondria to the anterograde motor kinesin-1 [28], whilst numerous adaptor proteins are thought to be involved in binding mitochondria to dynein for retrograde transport [4], although the precise mechanisms for mitochondrial tethering remain unclear. In retrograde transport, the function of dynein is dependent on the dynactin complex (includes proteins encoded by *DCTN 1/2*) as it links cytoplasmic dynein to its cargo and regulates dynein activity [25, 27]. Additionally, the activity of kinases, phosphatases, Rab-GTPases and Ca^2+^ concentrations also regulate the cargo-motor associations [14].

## Dysregulation of the intracellular transport machinery and mitochondrial function in ALS and other neurodegenerative diseases

In the context of aging and neurodegeneration, pathological structural changes and/or suboptimal functioning of the CNS are well established [29–32]. However, the specific mechanisms, particularly the molecular pathways that contribute to such changes are not completely understood and require further investigation. Disrupted axonal trafficking is a known feature of many neurodegenerative diseases, including ALS, PD, AD, HD, and Charcot-Marie-Tooth (CMT) disease. For instance, the initial segment of axons on motor neurons from individuals with ALS is swollen and contains accumulations of vesicles, lysosomes, mitochondria and intermediate filaments, including neurofilaments [15]. These observations are indicative of axonal transport and cytoskeletal defects. In addition, there is substantial evidence for mitochondrial dysfunction in ALS including dysregulation of mitochondrial proteins, increased production of mitochondrial reactive oxygen species (mtROS), decreased production of ATP and compromised mitochondrial quality control, which are likely to further exacerbate the axonal transport disruptions [33, 34]. However, whether these are major causative or contributing factors, or merely a consequence of neuronal degeneration, remains unclear. Axonal transport deficits may arise from various sources, including defects of cytoskeletal organization, impairment of motor protein attachment to microtubules, altered kinase activities, destabilization of motor-cargo binding, energetic mitochondrial breakdown, and/or dysregulated autophagic processes [15, 35]. In part, these aforementioned mechanisms may arise from mutations in gene loci encoding proteins for axonal transport structures or machinery that have been linked to the pathogenesis of neurological diseases [23]. Below we will discuss known genetic loci that affect the assembly and/or structural integrity of neurofilaments and microtubule network, thereby contributing to compromised axonal transport in neurodegenerative diseases.

### Genomic variants that contribute to cytoskeletal dysregulation in ALS

In ALS and other neurodegenerative diseases such as PD, AD and CMT, a common pathological hallmark is the formation of neuronal cytoplasmic inclusions containing intermediate filament proteins such as NEFL, NEFM, NEFH and PRPH [36–38]. Mislocalization of TAR DNA binding protein 43 (TDP-43) is believed to drive this pathology specifically in ALS [39], which will be discussed later. Besides TDP-43 mislocalization, some known mutations across the coding regions and intron–exon boundaries of neurofilament genes can also contribute to this pathology.

While mutations in *NEFL* are not reported as a primary cause for ALS, a rare polymorphism in the tail domain of NEFL was found in a single patient with sporadic ALS and in a family with CMT type 2, indicating that this variant is benign or that the pathogenicity of this variant may be dependent on other genetic factors [40]. On the other hand, other mutations in the head and rod domains of NEFL are associated with CMT type 1 and 2 [41, 42]. In CMT, *NEFL* mutations influence the propensity of cultured cells to form neurofilament aggregates [43] and also disrupt neurofilament assembly [44]. Two *NEFM* polymorphisms have been identified in one sporadic ALS and one familial ALS patient and may confer increased risk of ALS [40]. Mutations in the rod domain of NEFM have also been associated with PD [45] and AD [46], occurring in the protein domain that is critical for neurofilament assembly. Mutations across three protein domains (head, rod and tail) of NEFH are all associated with ALS [47–49]. Importantly, deletions or insertions in the tail region of *NEFH* result in changes in the phosphorylation domain and have a significant impact on the maintenance, assembly and transport of the neurofilaments [38, 47–50]. Several variants have also been reported in *PRPH* that are associated with ALS risk [51, 52]. In particular, a frameshift deletion reported in exon 1 results in a truncated PRPH protein that disrupts the assembly of intermediate filament structures in SW13 cells [52]. Another factor that may contribute to the neurofilament aggregation in ALS, particularly in sporadic ALS where no known genetic mutations have been identified, is the reduction of mRNA levels of the smaller neurofilaments *NEFL*, *PRPH* and *INA,* which in turn affects the ability of neurofilaments to assemble correctly [53–55].

While it is clear that the altered levels of intermediate filament-encoding mRNAs are critical in the pathogenesis of ALS, microRNAs (miRNAs) may also play a role in this differential expression pattern. Studies have shown that several miRNAs (miR-105 and miR-9) are dysregulated in the spinal cord of ALS patients and can directly contribute to the stability and expression of *NEFL*, *PRPH* and *INA* transcripts [55]. In addition, miRNAs that directly regulate *NEFL*, *NEFM* and *NEFH* transcripts are also dysregulated in ALS patients [54, 56]. Therefore, targeting miRNAs that disrupt the stoichiometry of intermediate neurofilament mRNAs may represent a valuable therapeutic strategy to reduce ALS pathology in motor neurons. In addition, it is known that non-coding genetic variation can influence the regulation of gene transcripts and may explain the lack of heritability for sporadic ALS [57]. Therefore, structural variation in neurofilament and other cytoskeletal genes could contribute to the changes in mRNA and protein stoichiometry observed in neurodegenerative diseases.

The disorganization of microtubules as observed in ALS may be attributed to several mutations and rare burden variants in alpha-tubulin 4a (encoded by *TUBA4A*) [58]. These variants have been reported as a cause for ALS [58]. Although *TUBA4A* is widely expressed, its expression is highest in the CNS and increases with age [58], potentially explaining why mutant *TUBA4A* causes disease in older individuals. The reported variants demonstrate impaired dimerization and decreased incorporation into microtubules in vitro, thereby destabilising the microtubule network and preventing its polymerization capacity [58]. Furthermore, the expression of genes that encode alpha-tubulin and microtubule-associated genes is downregulated in the spinal cord of individuals with sporadic ALS [57]. Mutations described to cause neurodevelopmental (i.e. cortical malformations) and neurodegenerative disorders have also been identified in seven other tubulin family members, including *TUBA1A* [59, 60], *TUBA8*, *TUBB2B* [61], *TUBB3* [62, 63], *TUBB4A* [64], *TUBB5* [65] and *TUBG1* [66].

### Genomic variants that contribute to molecular motor dysfunction in ALS

Both kinesin and dynein are ATP-dependent molecular motors and have a catalytic motor domain that binds directly to microtubules and generates movement through ATP hydrolysis [67, 68]. Since mitochondria can produce large amounts of ATP, they can directly influence the axonal transport of various cargos, including mitochondria themselves. The anterograde movement of the mitochondria is enabled by KIF5A *via* its C-terminal interaction with the adaptors Trak1 and Miro1/2 [69]. Mutation of *KIF5A* in zebrafish reduces both the number and the velocity of anterogradely moving mitochondria and results in a deficit of axonal mitochondria [69]. In addition to the role in fast axonal transport of mitochondria, KIF5A is also involved in the slow axonal transport of neurofilament proteins. Mice lacking *KIF5A* exhibit accumulation of neurofilaments in the cell body of peripheral sensory neurons, causing axonal reductions, loss of large-calibre axons and degeneration [14]. Importantly, mutations in *KIF5A* have been identified in ALS, frontotemporal dementia (FTD), hereditary spastic paraplegia and rare cases of CMT disease type 2 [70–74].

Conversely, it is well known that the dynein-dynactin motor complex plays a critical role in the retrograde transport of organelles and proteins, including mitochondria and neurofilaments, neurotrophic factors, endosomes and lysosomes, misfolded proteins for degradation and injury signals. Cultured dorsal root ganglion neurons, which express mutant dynein, encoded by *DYNC1H1,* exhibit impaired retrograde transport of mitochondria, resulting in increased cell death [75]. Similarly, mice with mutant *DYNC1H1* show significant retrograde transport defects and develop sensory neuropathy and loss of motor function [76]. Mutations in *DYNC1H1* have been identified in patients with CMT and spinal muscular atrophy [77, 78]. In addition, polymorphisms in dynactin, encoded by *DCTN1,* have been reported in ALS, PD, AD and FTD [79, 80]. However, variants in *DCTN1* do not segregate families with disease, indicating that they are not a primary cause for ALS, PD and FTD risk and potentially have either low disease penetrance or instead act as disease modifiers [79]. Interestingly, an individual with slowly progressive, chronic axonal distal motor neuropathy and the extrapyramidal syndrome has been reported to carry variants in *DCTN1*, *KIF5A* and *NEFH* genes [81].

Taken together, these findings demonstrate that mutations/variation in key genes involved in cytoskeletal organization and axonal transport can have a detrimental impact on structural organization, resulting in disrupted mitochondrial transport in neurons. Beyond the mutations in the structural components of the cytoskeleton, there are many other supporting proteins that facilitate axonal transport. The involvement of axonal transport-supporting proteins in neurodegenerative diseases has been reviewed [10].

### Abnormal protein activities that contribute to cytoskeletal dysregulation and mitochondrial dysfunction in ALS

In neurodegenerative disorders, protein abnormalities are commonly the result of altered conformational states, with underlying causes likely related to faulty trafficking and mislocalization of proteins and/or faulty protein quality control. Conformational changes like those observed in neurodegenerative disorders give rise to proteins with altered properties, which promote their aberrant accumulation and aggregation, and disrupt their interactions with other proteins.

As mentioned previously, cytoplasmic mislocalization and aggregation of TDP-43 is an established pathologic feature in around 97% of cases of ALS and frontotemporal lobar degeneration (FTLD) [82]. Interestingly, transgenic mice expressing ALS-linked TDP-43 mutants exhibit brain and spinal cord aggregates of PRPH, NEFH and NEFM proteins [83]. In addition, similar to the motor neurons of individuals with sporadic ALS [53], these animals have reduced levels of NEFL [83]. TDP-43 aggregation also increases NEFH protein levels in the cell body, which is followed by microglial recruitment to the affected area in a TDP-43 mislocalization mouse model [39]. Importantly, another recent study in transgenic ALS/FTD mice expressing hTDP-43 mutants has shown that the mislocalized TDP-43 represses the translation of neurofilament mRNAs (NEFL, NEFM and INA), giving rise to a proteomic signature functionally associated with cytoskeletal disorganization [84]. Treatment of these animals with an inhibitor of NF-kB signaling reduces inflammation and the level of TDP-43 aggregation, while increasing autophagy markers, restoring the translation of neurofilaments and reversing neuronal damage [84]. The loss of TDP-43 also reduces both the transcript levels and synthesis of neurofilament proteins, as well as the number and function of mitochondria in the axons of mouse motor neurons [85]. Together, these findings indicate that TDP-43 mediates dysregulated turnover and altered availability of NEFL, PRPH and other intermediate filaments, likely altering the stoichiometry of the neurofilaments, and in turn prevents their correct assembly [37], resulting in the formation of inclusions and large axonal swellings (spheroids) that further disrupt axonal transport [86].

Cytoplasmic TDP-43 can also enter mitochondria, where it is found at elevated levels in the cortex of FTD patients and the spinal cord of ALS patients [87]. In the mitochondrial matrix, TDP-43 binds to the mitochondria-transcribed mRNAs which encode the respiratory complex I subunits ND3 and ND6 and represses their translation, resulting in disrupted complex I activity, reduced ATP synthesis, loss of mitochondrial membrane potential and increased production of mtROS [87–89]. These aberrations in mitochondria culminate in the dysfunction and degeneration of neurons [87–89]. Recently, it has been demonstrated that the intramitochondrial TDP-43-induced production of mtROS also triggers the opening of the mitochondrial permeability transition pore and leakage of mitochondrial DNA into the cytoplasm, resulting in the activation of the cyclic guanosine monophosphate-adenosine monophosphate synthase (cGAS)/stimulator of interferon genes (STING) pathway in ALS patient iPSC-derived motor neurons and the spinal cords of transgenic mice expressing ALS-associated TDP-43 [90]. STING in turn activates the NF-kB and type I interferon (IFN-I) signaling pathways, which are also elevated in ALS patients [90]. These findings suggest a link between TDP-43 proteinopathy and the cGAS/STING pathway, which likely drives the neuroinflammatory process present in ALS and may also influence the progression of TDP-43-driven neurodegeneration. This pathway has also been implicated in several other chronic CNS pathologies [91], such as PD [92]. Notably, mitochondrial dysfunction and impaired energy production are also apparent in motor neurons that lack TDP-43 and treatment of these cells with the NAD + precursor nicotinamide rescues axonal growth [85]. Taken together, these findings indicate that the aberrant TDP-43 activity is likely to be a key link between cytoskeletal integrity, mitochondrial function and downstream inflammatory pathways.

In the context of protein trafficking in healthy neurons, the localization of neurofilament proteins is critical as they provide structural support and regulate axonal diameter, facilitating appropriate conduction velocity [93, 94]. Neurofilament proteins are synthesized in the cell body and are subsequently translocated to axons for assembly. Once in the axon, they undergo selective phosphorylation, which is a prominent regulatory mechanism and determining factor for proper localization [17]. As described above, the head domains of neurofilaments are phosphorylated in the cell body of neurons so that they remain disassembled for transport along the microtubules [18]. On the other hand, the tail region of neurofilament proteins is not phosphorylated in the cell body and dendrites. However, the tail regions of NEFM and NEFH proteins contain a polymorphic region consisting of amino acid repeats of lysine-serine-proline, known as the KSP repeat domain, which is extensively phosphorylated as NEFM and NEFH are transported along the neurofilament in the axon [3]. As these filaments move towards the distal end of the axon, the degree of phosphorylation in the KSP region correlates with the reduced transport velocity so that they eventually halt and are included in a pool of nearly immobilized axonal neurofilaments [95]. While genomic variants that alter the neurofilament phosphorylation in ALS have been described above, the activity and regulation of kinases that are responsible for this selective phosphorylation may also play an important role in maintaining the structural integrity of the cytoskeleton. Indeed, neurofilaments are a substrate for protein kinase N1 (PKN1), a serine/threonine kinase that phosphorylates the head-rod domain of neurofilament proteins. High levels of active PKN1 disrupt neurofilament assembly and their axonal transport, giving rise to focal aggregates of neurofilaments in neuronal cell bodies [96, 97]. Since neurofilament phosphorylation is regionally and temporally regulated by a balance of kinase and phosphatase activities [98], dysregulation of this balance likely contributes to ALS. Consistent with this notion, induction of glutamate excitotoxicity, a major pathway involved in ALS, induces caspase-mediated cleavage of the inhibitory N-terminal regions of PKN1, triggering the constitutive activation of PKN1 and neurofilament disruption in primary rat neurons [97]. Similarly, in the SOD1-G93A mouse model of ALS, PKN1 is cleaved and subsequently disrupts neurofilament organization and impairs axonal transport [97].

Stathmin-2 (STMN2) is one of the most abundantly expressed genes in motor neurons [99] and plays a critical role in the rapid polymerization of microtubules by regulating the assembly and catastrophe of alpha- and beta-tubulin [1, 100]. Following axonal injury, STMN2 expression is increased and is anterogradely transported toward the growth cones of regenerating axons [101]. STMN2, therefore, functions as a critical axonal maintenance factor that can accelerate neurodegeneration when dysregulated [99]. Interestingly, levels of STMN2 are directly influenced by the RNA-binding protein TDP-43, which binds to intron 1 of *STMN2* pre-mRNA and suppresses the inclusion of a cryptic exon, thereby preventing premature polyadenylation and missplicing of *STMN2* [99]. Disruption of TDP-43 in individuals with sporadic and *C9orf72* ALS, or in cultured human neurons with ALS-causing TDP-43 mutations, gives rise to a truncated, non-functional mRNA that undergoes nonsense-mediated decay [99, 102]. As a result, expression of functional STMN2 is decreased in motor neurons from individuals with ALS, contributing to impaired axonal outgrowth [99, 102]. Furthermore, Klim et al. [102] have demonstrated that phosphorylation of STMN2 by mitogen-activated protein kinase-8 (MAPK8, encoded by *JNK1*) is also implicated in ALS [102]. The MAPK8-mediated phosphorylation of STMN2 reduces its activity and promotes its degradation [103, 104]. Remarkably, inhibition of MAPK8 increases STMN2 levels and rescues axonal outgrowth, even in states of TDP-43 depletion, thus improving the microtubule stability and transport networks in human embryonic stem cell-derived motor neurons [102]. These important studies demonstrate that the abnormal interactions between TDP-43 and STMN2, as well as the post-translational modification of STMN2 by MAPK8, are likely to contribute to the motor neuron dysfunction and degeneration in ALS. Truncated STMN2 has also recently been identified as a pathologic feature of PD and FTD [105, 106], highlighting a potential common pathway leading to neurodegeneration. In addition, a polymorphism in the *STMN2* gene has recently been associated with disease risk, age of onset, disease progression and survival of bulbar-onset sporadic ALS patients [107]. Although further validation is required, this is the first report of a genetic link between *STMN2* and sporadic ALS.

### Dysfunctional mitochondrial quality control that contributes to impaired axonal transport in ALS

Functional mitochondria are required to provide adequate energy to support healthy axonal functions over large distances [108]. Mitochondria constantly change their morphology and localization in cells under given conditions to maintain homeostasis. The homeostasis of mitochondria is dynamically regulated by a balanced and continuous cycle of fusion, in which defective mitochondria are fused with healthy mitochondria to enable repair of damaged mitochondrial components, and fission, which segregates and eliminates defective mitochondria [109]. Mitochondrial fusion is mediated by mitofusin (encoded by *MFN1/2*) and optic atrophy-1 (encoded by *OPA1*), while mitochondrial fission is mediated by dynamin-related protein-1 (encoded by *DRP1*) and fission-1 (encoded by *FIS1*) [109]. Thus, any disturbance affecting mitochondrial integrity, morphology, or the dynamic balance between mitochondrial fission and fusion influences axonal transport [14]. Interestingly, *FIS1* interacts with the ALS-linked *C9orf72* gene, as recently examined in a synthetic lethal screen [110]. Mutations in *FIS1* are disruptive to the proper disposal of defective mitochondria [111], indicating that FIS1 is able to direct mitochondria towards degradation and to couple stress-induced mitochondrial fission with downstream degradation processes. This occurs by the formation of a complex with DRP1 and endoplasmic reticulum (ER) proteins, which acts as an interface between the mitochondria and ER. DRP1 is implicated in the pathogenesis of multiple neurodegenerative diseases such as ALS, AD, HD and PD [112–114], where dysregulation of DRP1 is associated with changes in mitochondrial morphology and decreased energy production in axons.

Mitochondrial uptake of Ca^2+^ is required for correct intracellular signaling, homeostasis and mitochondrial integrity and transport. Calcium fluxes between the cell and the extracellular matrix, between the cytosol and intracellular Ca^2+^ depots (mitochondria and ER), as well as between the depots themselves are essential for proper neuronal function. Mitochondrial distribution can therefore be influenced by the ER, although the mechanism is only partially understood [115]. The interactions between the resident ER protein, vesicle-associated membrane protein-associated protein-B (VAPB), and the outer mitochondrial membrane protein, protein tyrosine phosphatase-interacting protein-51 (PTPIP51), is demonstrated to regulate the ER-mitochondria associations and calcium homeostasis in neurons [116, 117]. Increased expression of wild-type or ALS-associated mutants of TDP-43 or fused in sarcoma (FUS) protein activates the glycogen synthase kinase-3β (GSK-3β), which in turn prevents VAPB and PTPIP51 from interacting [117, 118]. The ensuing disruption of the interaction between ER and mitochondria increases cytosolic Ca^2+^ levels and deregulates mitochondrial Ca^2+^ levels and ATP synthesis [117, 118]. The increased cytosolic Ca^2+^ levels inhibit mitochondria motility [2]. Importantly, a mutation in VAPB causes familial ALS and disrupts the anterograde transport of mitochondria [119]. In VAPB^P56S^ neurons, cytosolic Ca^2+^ levels are increased, halting mitochondrial transport by triggering the release of adaptor Miro1 and its associated anterograde motor kinesin-1 from microtubules [119]. Taken together, these findings indicate that the dysregulation of Ca^2+^ homeostasis is a central disruptor of axonal transport and mitochondrial health in motor neurons during ALS.

Mitochondrial quality control is central to the health of cells and is achieved through a process known as mitophagy. During mitophagy, stressed or damaged mitochondria that cannot be rescued by fusion/fission cycling are selectively targeted for degradation *via* autophagy. Mitophagy is primarily mediated by the PTEN-induced kinase 1 (PINK1)/E3 ubiquitin ligase (PARKIN) pathway. Active PINK1 promotes degradation of kinesin-associated Miro, which arrests anterograde mitochondrial transport [120, 121]. Full-length PINK1 is demonstrated to facilitate retrograde transport, however, PINK1 is also able to promote degradation of dynein-associated Miro and inhibit transport altogether [120, 121]. In healthy conditions, the high mitochondrial membrane potential triggers the import and proteolysis of PINK1 by intra-mitochondrial proteases, leading to its ubiquitination and degradation [122]. However, in stress or damage, mitochondrial depolarization prevents the import of PINK1 and instead, the full-length protein accumulates in the cytoplasm at the outer mitochondrial membrane, where it undergoes dimerization and phosphorylation [122]. The activated PINK1 initiates a positive feedback loop of phosphorylation and ubiquitination by phosphorylating PARKIN, activating its ubiquitin E3 ligase function so that it can ubiquitinate substrates on the outer mitochondrial membrane [122]. The phospho-ubiquitin chains on the outer membrane of dysfunctional mitochondria activate and recruit a cascade of adaptor proteins, including tank binding kinase-1 (TBK1), which phosphorylates optineurin (OPTN), sequestosome-1 (SQSTM1, p62) and nuclear dot protein-52 (NDP52), thereby associating damaged mitochondria with microtubule-associated proteins 1A/1B light chain 3 (LC3) to initiate mitophagy [122]. Evidently, faulty quality control of mitochondria is an emerging feature of ALS, with many familial forms of the disease arising from mutations in key regulators of mitophagy, such as TBK1, OPTN and p62 [123–125]. Furthermore, OPTN is present in TDP-43-positive aggregates in patients with sporadic ALS and in SOD1-immunopositive inclusions in individuals with SOD1 familial ALS, suggesting that OPTN may be involved in the broader pathogenesis of ALS [123, 126, 127]. Similarly, in FTLD with ALS (FTLD-ALS) patients, p62 is also sequestered into cytoplasmic TDP-43-positive inclusions throughout the CNS [126, 127]. Taken together, these findings suggest that the dysfunction or sequestration of mitophagy-associated proteins likely allows severely damaged, dysfunctional mitochondria to accumulate in motor neurons and contribute to disease in patients with ALS.

It is notable that in addition to its critical function in surveillance pathways of mitochondrial health, PINK1 also plays a role in facilitating mitochondrial transport. PINK1 is capable of forming a protein complex with the adaptor proteins Miro and Milton, which anchors mitochondria to the anterograde motor kinesin-1 [28]. In contexts of mitochondrial stress or damage, activated PINK1 targets Miro for proteasomal degradation [120, 121]. The destruction of Miro detaches kinesin from the mitochondrion and arrests its movement [120]. Furthermore, PINK1 is demonstrated to act as a molecular switch between anterograde and retrograde mitochondrial transport [128]. While the cleaved form of PINK1 in healthy mitochondria promotes anterograde transport, the full-length protein promotes retrograde transport [128]. Since the full-length PINK1 is associated with defective mitochondria, these findings are consistent with the movement of damaged mitochondria towards the lysosome-rich perinuclear area for mitophagy [128]. Interestingly, the activity of both cleaved and full-length PINK1 in axonal transport is enhanced when phosphorylated at T313 by microtubule affinity regulating kinase-2 (MARK2) [128]. Mutations at T313 in PINK1 commonly occur in familial PD [129], suggesting that disruptions in microtubule stability and mitochondrial transport may be the result of faulty PINK1/MARK2 signaling.

The disruption of PINK1 signaling also has precedence as a contributing factor in ALS. PINK1 and PARKIN are dysregulated in SOD1-G93A mice and in individuals with sporadic ALS [130, 131]. Furthermore, overexpression of TDP-43 reduces PARKIN levels and impairs proteasomal activity, promoting the accumulation of both full-length and cleaved PINK1 in insoluble cytoplasmic aggregates [132]. Similarly, mice that express ALS-associated mutant TDP-43 have decreased levels of PARKIN and increased levels of full-length and cleaved PINK1 protein in the CNS [132]. The accumulation of cleaved PINK1 is correlated with impaired mitochondrial function and is therefore likely to contribute to disease in TDP-43-mediated proteinopathy [132]. Similarly, enhanced expression of wild-type or ALS-associated FUS mutants promotes the aggregation of PINK1 and PARKIN proteins, as well as the ubiquitination of Miro1 [133]. Accordingly, axonal transport of mitochondria is disrupted by FUS, particularly in the case of an ALS-associated mutant [133]. Of interest, retrograde mitochondrial transport is more severely affected by FUS [133]. Since damaged mitochondria are normally transported towards the lysosome-rich perinuclear area for mitophagy, impaired retrograde transport is likely to give rise to an accumulation of damaged, dysfunctional mitochondria in the distal axons, which promotes motor neuron dysfunction. Taken together, the PINK1/PARKIN signaling represents an interesting intersection between mitochondrial quality control and axonal transport in ALS and PD. The precise nature of this intersection requires further clarification and should be the target of further research efforts.

## Emerging mediators of cytoskeletal and mitochondrial dysfunctions in ALS

An important question remains: does the neurodegenerative pathology in ALS originate in the cell body, where protein aggregates impair axonal trafficking of mitochondria and other cargos, causing the ATP-starved distal termini of motor neurons to degenerate? Alternatively, does neurodegeneration originate as a result of impaired trafficking of toxic metabolites from the termini of motor neurons towards the cell body for removal? There is evidence for both and these pathological processes are not necessarily mutually exclusive.

**Fig. 1:**
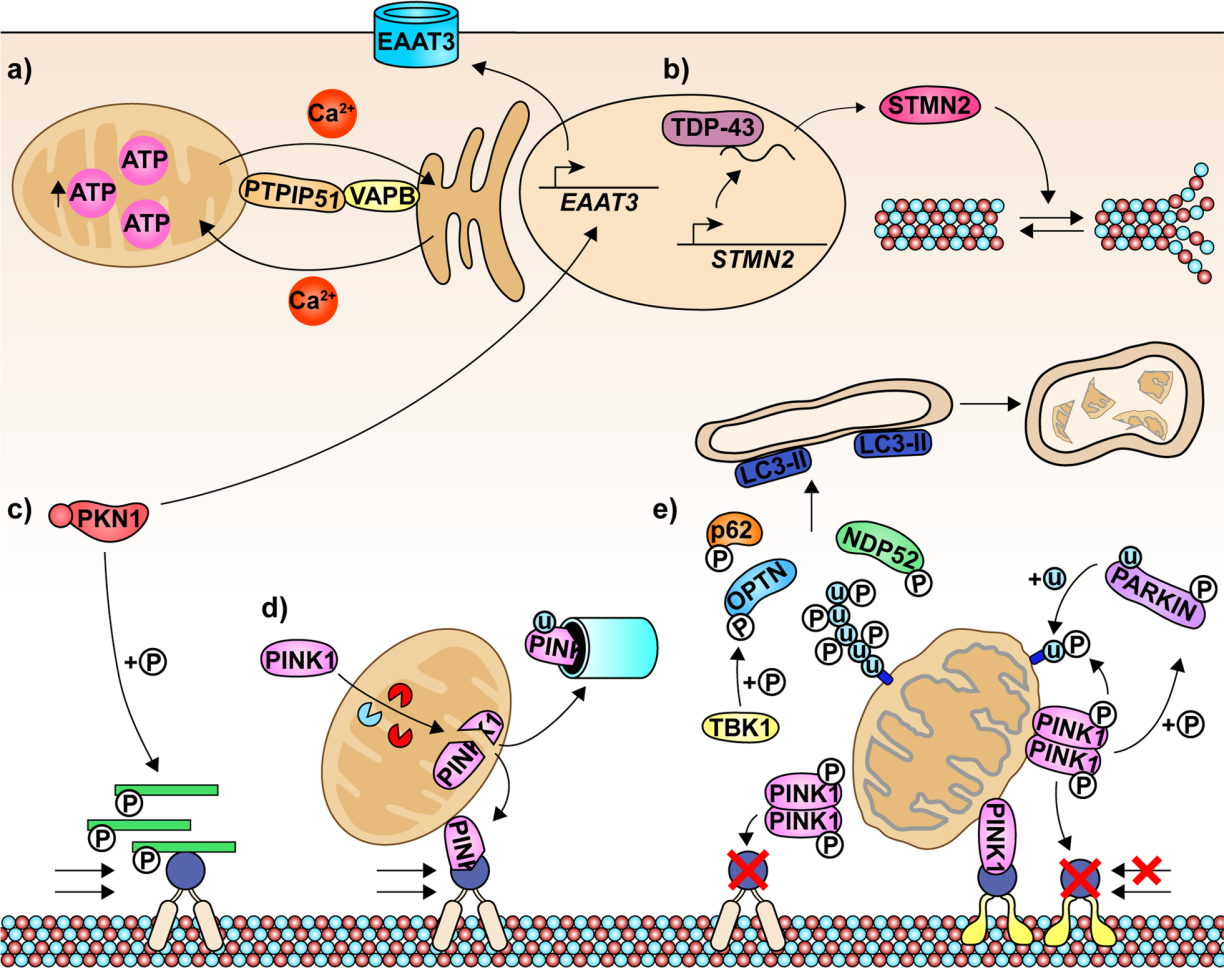
Selected aspects of cytoskeletal transport and mitochondrial dynamics during homeostasis. **a** The interaction between ER-bound VAPB and mitochondria-bound PTPIP51 allows calcium ions to cycle between these organelles in order to maintain mitochondrial integrity, homeostasis and ATP production. **b** Association of TDP-43 with the pre-mRNA of STMN2 prevents the inclusion of a cryptic exon. As a result, full-length, mature STMN2 is produced, which modulates the polymerization and disassembly of microtubules. **c** The kinase PKN1 phosphorylates the head-rod domain of neurofilaments, which prevents these filaments from forming dimers so that they can be transported towards the distal end of the axon. PKN1 also elevates the expression of neuronal glutamate transporters, such as EAAT3. **d**, **e** PINK1/PARKIN signaling acts as a molecular switch between anterograde and retrograde mitochondrial transport. **d** In healthy mitochondria, PINK1 is imported and cleaved by intramitochondrial proteases. The cleaved PINK1 is degraded by the proteasome but is also demonstrated to bind to Miro-kinesin complexes and facilitate anterograde transport. **e** On the other hand, PINK1 is not imported into damaged, depolarized mitochondria and is instead activated through dimerization and autophosphorylation. The active PINK1 promotes the degradation of kinesin-associated Miro, which arrests anterograde mitochondrial transport. Full-length PINK1 is demonstrated to facilitate retrograde transport; however, PINK1 is also able to promote degradation of dynein-associated Miro and inhibit transport altogether. Active PINK1 phosphorylates PARKIN. Together, these proteins phosphorylate and ubiquitinate proteins on the mitochondrion surface. The phospho-ubiquitin chains activate TBK1, which in turn phosphorylates and activates mitophagy adaptor proteins, such as OPTN, p62 and NDP52, which initiate the removal of damaged mitochondria through the process of mitophagy

**Fig. 2:**
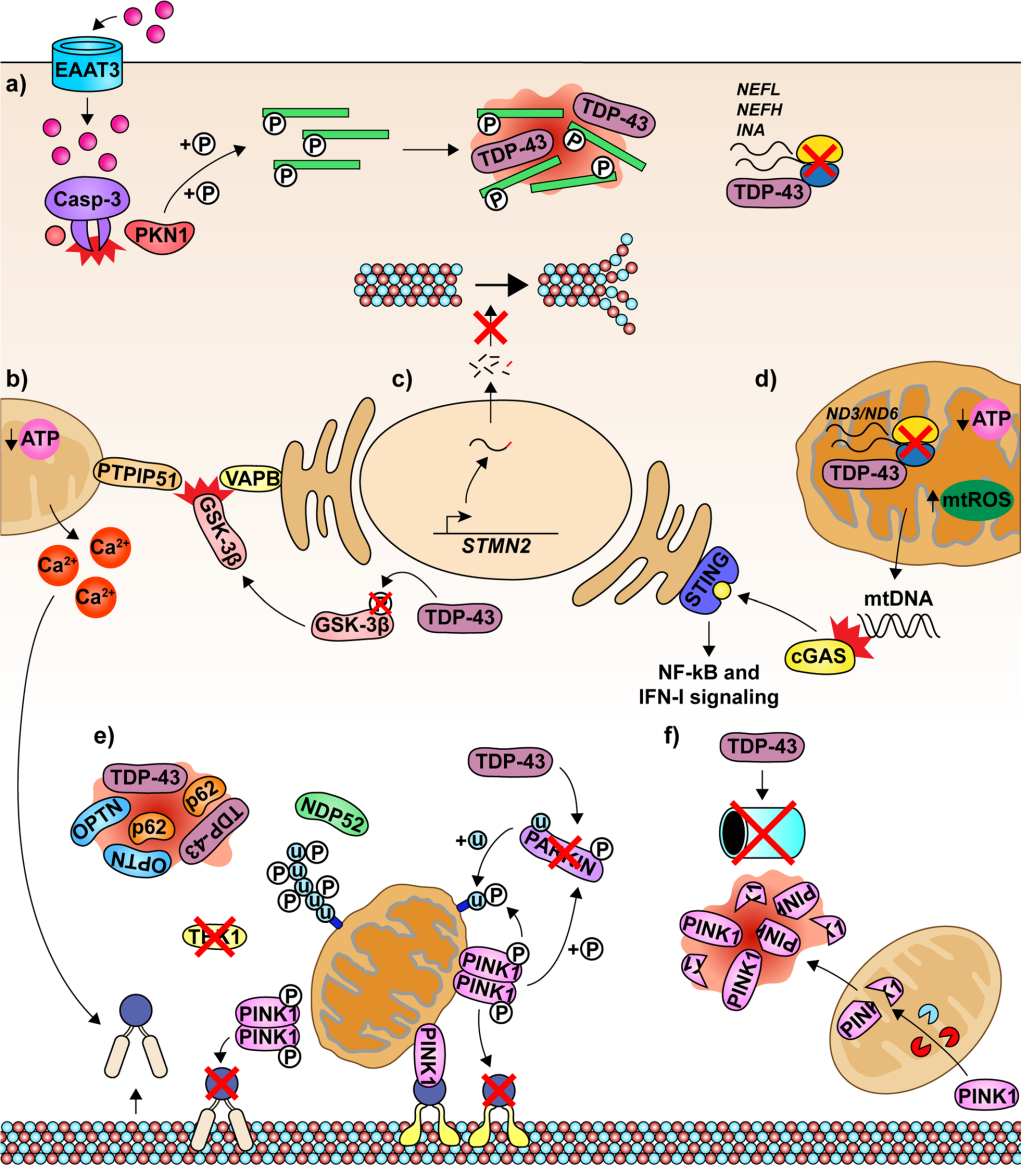
Emerging mediators of cytoskeletal and mitochondrial dysfunction in ALS. In 97% of ALS and FTLD cases, TDP-43 is mislocalized to the cytoplasm, where it causes significant disruption to the homeostatic functions of neurons. **a** Exposure of neurons to toxic levels of excitatory neurotransmitters, as observed in ALS, triggers caspase-mediated cleavage of the inhibitory domain of PKN1. Constitutive activation of PKN1 is pathologic and causes aberrant phosphorylation of neurofilaments, which accumulate in TDP-43-positive aggregates. Mislocalized TDP-43 also represses the translation of neurofilament mRNAs. These aberrations disrupt the correct stoichiometry of neurofilaments and prevent the correct assembly of neurofilament structures. **b** Cytoplasmic TDP-43 activates GSK-3β, which blocks the interaction between VAPB and PTPIP51. The disruption of the ER-mitochondria calcium cycling reduces mitochondrial Ca^2+^ levels, which impairs ATP synthesis and increases cytosolic Ca^2+^ levels,  resulting in removal of the Miro-kinesin complexes from microtubules and inhibition of mitochondrial motility. **c** In the absence of nuclear TDP-43, truncated STMN2 mRNA is produced and undergoes nonsense-mediated decay. The loss of STMN2 contributes to impaired axonal outgrowth. **d** Cytoplasmic TDP-43 enters the mitochondria and represses translation of respiratory complex I mRNAs encoding ND3 and ND6, causing mitochondrial dysfunction, production of mtROS and leakage of mitochondrial DNA (mtDNA). The cytoplasmic mtDNA activates the cGAS/STING pathway and triggers NF-kB and IFN-I signaling. **e** TDP-43 also disrupts PARKIN levels, limiting the ubiquitination of surface proteins on damaged mitochondria. Furthermore, dysfunctional TBK1 is implicated in certain subtypes of ALS, while mitophagy adaptor proteins such as OPTN and p62 are sequestered in TDP-43 aggregates in individuals with ALS or FTLD-ALS. In all, these disruptions are likely to impair mitophagy and give rise to the accumulation of severely damaged, dysfunctional mitochondria, which disrupt motor neuron functions in patients with ALS. **f** Mislocalized TDP-43 impairs proteasomal function. As a result, full-length and cleaved PINK1 form insoluble cytoplasmic aggregates

Mutations have been reported in many different genes involved in both anterograde and retrograde transport in ALS, PD, AD, HD and CMT [15]. What is clear in the literature is that mitochondria can accumulate in the cell body and the proximal or distal parts of the axon, under circumstances of neurodegeneration [134]. We have reviewed and discussed certain aspects of the delicate balance between mitochondrial dynamics (for maintaining adequate energy production and mitochondrial integrity, and facilitating transport) and cytoskeletal integrity (for maintaining transport networks and axonal diameter) during homeostasis (Fig. 1), as well as its dysregulation in neurodegenerative diseases such as ALS (Fig. 2). In properly deciphering this balance, there remains much to be uncovered. Evidently, it is important to explore new molecular pathways that may underpin this relationship and give insight into how all aspects of cytoskeleton disruption contribute to mitochondrial mislocalization.

### A potential role for PKN1 in cytoskeletal and mitochondrial disruption in ALS

Here we suggest a potential role for PKN1 that might be applicable to disruptions in retrograde transport. PKN1 has a catalytic domain homologous to the domain in protein kinase C [135], a known inhibitor of retrograde transport [136]. This homology suggests that PKN1 may be another disrupting factor in retrograde trafficking. This is significant because damaged protein structures, such as neurotransmitter receptors, organelles and, most notably, mitochondria, are subject to retrograde transport away from axonal termini and toward the soma for endolysosomal degradation. Importantly, PKN1 has also recently been recognized as a key regulator of synaptic maturation and synaptic transmission, suggesting that PKN1 may play a more central role in neuronal homeostasis than previously thought [137]. PKN1 elevates the expression of neuronal glutamate transporters such as excitatory amino acid transporter-3 (EAAT3) and increases the activity of developing neurons [137]. However, as described above, exposure of neurons to excitotoxic levels of glutamate elevates the PKN1 activity and disrupts the axonal trafficking of neurofilaments [96, 97]. Taken together, these findings provide insight into an important connection between the role of PKN1 in the regulation of neuronal activity and its role in cytoskeleton integrity disruption. The dysregulated activity of PKN1, as observed in ALS, may contribute to the irreversible commitment to neurodegeneration* via* the disrupted retrograde transport of toxic compounds, cytoskeletal degeneration and/or dysregulated neuronal activity. At this point-of-no-return, the neuron may be unable to tolerate further consequences of glutamate receptor overstimulation, a common feature of neurodegenerative diseases, including ALS [138].

### Linking the gut microbiome to cytoskeletal and mitochondrial dysfunction in ALS

The microbiome consists of many microorganisms such as bacteria, viruses and fungi that live within the gut. The gut microbiome has been implicated in many neurodegenerative disorders through the bidirectional feedback system known as the gut-brain axis. In particular, the gut microbiota exists in a delicate balance, where dysbiosis can signal to the CNS through the production of neuromodulators such as tryptophan, choline and short-chain fatty acids [139]. The gut-brain axis has been predominantly explored in the context of PD and AD [140], however, emerging evidence also suggests a potential role in ALS that may influence the phenotypic variability between patients [141, 142].

Exploratory studies have shown mixed and contradictory findings in the microbial diversity, metabolite and cytokine levels between ALS patients and controls [141–143]. However, to date, sample populations have been relatively small and may reflect inter-group differences. Despite the variability reported in human studies, it has been shown that the ALS disease severity can be directly manipulated in antibiotic-treated SOD1-G93A mice by supplementing these animals with certain gut microbial species [144], highlighting the potential effect of gut metabolites on neural function. Of note, supplementation of antibiotic-treated SOD1 mice with *Akkermansia muciniphila* ameliorates symptoms and is associated with enrichment of gene pathways relating to mitochondria function and NAD + homeostasis [144]. Accordingly, treatment of SOD1 animals with the NAD + precursor nicotinamide recapitulates these findings, improving motor symptoms and neurological function in these mice [144]. In addition, individuals with ALS have decreased nicotinamide levels in the serum and cerebrospinal fluid compared with healthy controls [144]. These insights suggest that the microbiome-derived metabolites may regulate neuronal health by modulating mitochondrial activity and thus influence cytoskeletal integrity.

Other gut microbiome-generated metabolites, such as colonic acid and methyl metabolites, are able to pass the blood–brain barrier and modulate neuronal mitochondrial fusion-fission dynamics [145]. Additionally, bacterial populations responsible for producing the short-chain fatty acid butyrate have been shown to be significantly decreased in people with ALS compared with healthy controls [142, 146]. Importantly, short-chain fatty acids produced by the gut microbiome can up-regulate the peroxisome proliferator-activated receptor gamma coactivator 1-α (PGC-1α), a master regulator of mitochondrial biogenesis [147, 148]. This finding is intriguing, as the activity and levels of PGC-1α are dysregulated in individuals with ALS [109], and it is tempting to speculate that changes in the gut microbiome may be a contributing factor. Furthermore, the PKN family of kinases responsible for regulating cytoskeletal organization [135, 137] are activated by unsaturated fatty acids produced by the gut bacteria [149]. The composition of gut bacteria and the associated metabolic effects may therefore prove to be a significant effector of cytoskeletal organization in neurons during ALS, although the specifics of this interaction have yet to be explored.

## Concluding remarks

Although it is well-known that cellular dysfunctions that affect long-distance axonal transport and mitochondrial homeostasis likely contribute to neurodegenerative disorders, the links between these pathways have only recently begun to emerge. One such intersection linking both cytoskeletal and mitochondrial function is their respective interaction with TDP-43. In ALS, TDP-43 aggregation is seen in ~ 97% of cases, and recent evidence demonstrates that dysregulation of TDP-43 can drive changes in intermediate filament expression [83, 85], translation [84], localization [39] and stoichiometry, resulting in cytoskeletal disorganization [84]. In conjunction, mislocalized TDP-43 also directly causes mitochondrial dysfunction and triggers neuroinflammatory processes [87–90]. However, for the most part, the current understanding comes down to the association of 'key players' involved in facilitating axonal transport and mitochondrial dynamics. Therefore, further exploration of the intersection between cytoskeleton dysregulation and mitochondrial function is required to discern the precise mechanisms by which these pathways contribute to the pathology of ALS and other neurodegenerative diseases.

We believe that therapeutic approaches to maintaining and restoring the overall axonal integrity and mitochondrial localization and transport represent an important novel strategy that is urgently needed for the treatment of ALS and other neurodegenerative disorders. To date, pre-clinical animal models have been an invaluable tool to connect omics data and genetic information with clinical phenotypes in order to map relevant networks that are disrupted in neurodegenerative diseases. Using this information, it is important to examine the converging points of these networks (regulatory or otherwise) so that future therapeutic approaches can be evaluated within this context, thus enabling the development of targeted therapies.

## Data Availability

Not applicable.

## Abbreviations

ATP: Adenosine triphosphate
TUBA4A: Alpha-tubulin 4a
AD: Alzheimer's disease
ALS: Amyotrophic lateral sclerosis
CNS: Central nervous system
CMT: Charcot-Marie-Tooth disease
cGAS: Cyclic guanosine monophosphate-adenosine monophosphate synthase
DRP1: Dynamin-related protein-1
DYNC1H1 and DYNC1H2: Dynein motor complex heavy chains
ER: Endoplasmic reticulum
EAAT3: Excitatory amino acid transporter-3
PARKIN: E3 ubiquitin ligase
FIS1: Fission-1
FTD: Frontotemporal dementia
FTLD: Frontotemporal lobar degeneration
FUS: Fused in sarcoma protein
GSK-3β: Glycogen synthase kinase-3β
HD: Huntington's disease
INA: Internexin-alpha
LRRK2: Kinase leucine-rich repeat kinase-2
MARK2: Microtubule affinity regulating kinase-2
miRNAs: MicroRNAs
mtROS: Mitochondrial reactive oxygen species
MFN1 and MFN1: Mitofusin
MAPK8: Mitogen-activated protein kinase-8
NEFL: Neurofilament light
NEFM: Neurofilament medium
NEFH: Neurofilament heavy
NDP52: Nuclear dot protein-52
OPA1: Optic atrophy-1
OPTN: Optineurin
PD: Parkinson's disease
PRPH: Peripherin
PGC-1α: Peroxisome proliferator-activated receptor gamma coactivator 1-α
PKN1: Protein kinase N1
PINK1: PTEN-induced kinase 1
STMN2: Stathmin-2
STING: Stimulator of interferon genes
TDP-43: TAR DNA binding protein 43
TBK1: Tank binding kinase-1
PTPIP51: Tyrosine phosphatase-interacting protein-51
VAPB: Vesicle-associated membrane protein-associated protein-B
LC3: 1A/1B light chain 3
